# Mycobacterium abscessus Smooth and Rough Morphotypes Form Antimicrobial-Tolerant Biofilm Phenotypes but Are Killed by Acetic Acid

**DOI:** 10.1128/AAC.01782-17

**Published:** 2018-02-23

**Authors:** Gillian Clary, Smitha J. Sasindran, Nathan Nesbitt, Laurel Mason, Sara Cole, Abul Azad, Karen McCoy, Larry S. Schlesinger, Luanne Hall-Stoodley

**Affiliations:** a Department of Microbial Infection and Immunity, The Ohio State University College of Medicine, Columbus, Ohio, USA; b The Ohio State University College of Medicine, Columbus, Ohio, USA; c Department of Microbiology, Ohio State University, Columbus, Ohio, USA; d OSU Campus Microscopy and Imaging Facility, Ohio State University, Columbus, Ohio, USA; e Department of Pediatrics, Nationwide Children's Hospital, Columbus, Ohio, USA; f Texas Biomedical Research Institute, San Antonio, Texas, USA

**Keywords:** Mycobacterium abscessus, morphotype, biofilm, antibiotic tolerance, acetic acid

## Abstract

Mycobacterium abscessus has emerged as an important pathogen in people with chronic inflammatory lung diseases such as cystic fibrosis, and recent reports suggest that it may be transmissible by fomites. M. abscessus exhibits two major colony morphology variants: a smooth morphotype (*Ma^Sm^*) and a rough morphotype (*Ma^Rg^*). Biofilm formation, prolonged intracellular survival, and colony variant diversity can each contribute to the persistence of M. abscessus and other bacterial pathogens in chronic pulmonary diseases. A prevailing paradigm of chronic M. abscessus infection is that *Ma^Sm^* is a noninvasive, biofilm-forming, persistent phenotype and *Ma^Rg^* an invasive phenotype that is unable to form biofilms. We show that *Ma^Rg^* is hyperaggregative and forms biofilm-like aggregates, which, like *Ma^Sm^* biofilm aggregates, are significantly more tolerant than planktonic variants to acidic pHs, hydrogen peroxide (H_2_O_2_), and treatment with amikacin or azithromycin. We further show that both variants are recalcitrant to antibiotic treatment inside human macrophage-like cells and that *Ma^Rg^* is more refractory than *Ma^Sm^* to azithromycin. Our results indicate that biofilm-like aggregation and protracted intracellular survival may each contribute to the persistence of this problematic pathogen in the face of antimicrobial agents regardless of morphotype. Biofilms of each M. abscessus variant are rapidly killed, however, by acetic acid, which may help to prevent local fomite transmission.

## INTRODUCTION

Mycobacterium abscessus is a pathogenic nontuberculous mycobacterium (NTM) and the leading cause of infection attributed to rapidly growing mycobacteria (RGM). M. abscessus can cause skin and soft tissue infections in patients with healthy immune systems, as well as a variety of infections on medical implants (1–3). It has recently gained attention as the most common cause of RGM infections worldwide in people with chronic inflammatory lung diseases such as cystic fibrosis (CF), non-CF bronchiectasis, and chronic obstructive pulmonary disease (COPD), resulting in both nodular and cavitary granulomas and persistent lung infection (1, 2, 4–11). In contrast to the scenario for many NTM infections, antibiotic therapy often fails to lead to lasting sputum conversion (SC), and no antibiotic regimen reliably cures M. abscessus infection (1, 5, 12, 13). A recent systematic review of NTM pulmonary disease (PNTM) found that without adjunctive surgical resection, the rate of SC with medical treatment of M. abscessus infection was 34% (14). In studies that specifically determined results for M. abscessus sensu stricto, SC rates were even lower (25 to 32%). Mortality due to PNTM in the United States has increased by >8% per year and now outpaces mortality due to tuberculosis; PNTM fibrocavitary disease is associated with increased mortality (15, 16).

Pathogenic RGM, such as M. abscessus, Mycobacterium chelonae, and Mycobacterium fortuitum, are widely distributed in the environment, often in nutrient-poor, low-pH environments (17, 18). These NTM are also difficult to eradicate in nosocomial settings (19). We have shown previously that pathogenic RGM readily form aggregated structures (biofilms) by colonizing surfaces independently of other microorganisms (20, 21). Biofilms may contribute to the transmission of RGM by protecting bacteria from desiccation and by harboring high numbers of bacteria, which, if aerosolized, may lead to the inhalation of a condensed infective dose in aerosolized aggregates (22). Recent reports suggest that the increase in M. abscessus infections may be due to the global emergence of transmissible virulent clones that are possibly spread by aerosols or by fomites (23, 24). Understanding the virulence mechanisms of M. abscessus is therefore clinically relevant, particularly with regard to pulmonary infections.

Biofilm formation, extended intracellular survival, and colony variant diversification can each contribute to the persistence of select bacterial pathogens in CF (25–28). CF pathogens also share important traits in pathoadaptation to the CF airway, including antibiotic tolerance and evasion of innate immune effectors (25, 29, 30). For example, Pseudomonas aeruginosa, the most common pathogen causing lung infections in CF patients, forms biofilms that protect bacteria from antibiotic therapy and from effective host clearance during chronic lung infection (25, 29). P. aeruginosa colony morphology variants isolated from CF sputum include mucoid colonies and aggregative rugose small-colony variants, both of which are linked to extended antibiotic treatment and correlate with the onset of persistent infection (25, 30).

M. abscessus biofilm aggregates have recently been demonstrated in the lungs of patients with CF, non-CF bronchiectasis, and COPD (28, 31). M. abscessus exhibits two colony morphology variants: a smooth-colony variant (*Ma^Sm^*) that expresses glycopeptidolipid (GPL) on its cell wall and a rough-colony variant (*Ma^Rg^*) with diminished GPL expression on the cell surface (32–35). Both variants are found in patients with chronic lung infections; however, the *Ma^Rg^* variant is associated with more-aggressive pulmonary disease and is hypervirulent in a zebrafish infection model (10, 35–37). Previous research using an M. abscessus clinical isolate showed that a smooth variant formed biofilms, but a rough variant did not (32–35). These studies led to the proposition that GPL expression enhanced *Ma^Sm^* sliding motility in CF mucus and a colonizing, biofilm-forming phenotype, whereas *Ma^Rg^* was a non-biofilm-forming, invasive phenotype (32–34, 38, 39). According to this paradigm, persistent infection with M. abscessus is thought to be due to the transition (switching) between *Ma^Rg^* and *Ma^Sm^* colony variants (33, 34, 40). More recently genomic sequencing studies showing extensive disruption of the GPL locus in *Ma^Rg^* have cast doubt on the ability of *Ma^Rg^* to transition to a *Ma^Sm^* variant (35, 41). Furthermore, studies with many bacteria show that although biofilm phenotypes may be nonmotile, aggregation is necessary for antimicrobial tolerance (25, 26, 42–44).

Biofilms are most often described as assemblages of microbial cells that are attached to a surface. However, biofilms also form as suspended aggregates at air-liquid interfaces, and nonadherent aggregated bacteria show an antibiotic tolerance phenotype similar to that of adherent aggregates (42). In CF lung infections, the majority of aggregated P. aeruginosa bacteria are found within the inspissated mucus in larger airways rather than adherent to pulmonary epithelium (29). A consensus definition of biofilms as inherently anchored to a substratum has recently been modified to accommodate biofilm-associated infections (26, 45) and growing evidence that free-floating biofilm aggregates can profoundly affect the interplay between nutrient resources, spatial structure, bacterial fitness, and multicellular assembly (42, 44).

We hypothesized that M. abscessus antimicrobial recalcitrance was not restricted to one colony morphology variant, and we investigated each morphotype using isogenic *Ma^Sm^* and *Ma^Rg^* variants isolated from the sequenced M. abscessus ATCC 19977^T^ reference strain (46). Our results show that each M. abscessus colony variant formed biofilms that exhibited antimicrobial tolerance and that neither biofilm formation nor prolonged survival inside macrophages is morphotype restricted. *Ma^Rg^*, however, is more refractory than *Ma^Sm^* to antimicrobial treatment overall. We also show that although M. abscessus variants in biofilm-like aggregates are significantly more tolerant than planktonic bacteria to antimicrobial treatment, biofilms were rapidly killed with acetic acid, which may prevent the potential transmission of M. abscessus in clinical settings.

## RESULTS

### *Ma^Rg^* is more aggregative than *Ma^Sm^*.

**FIG 1:**
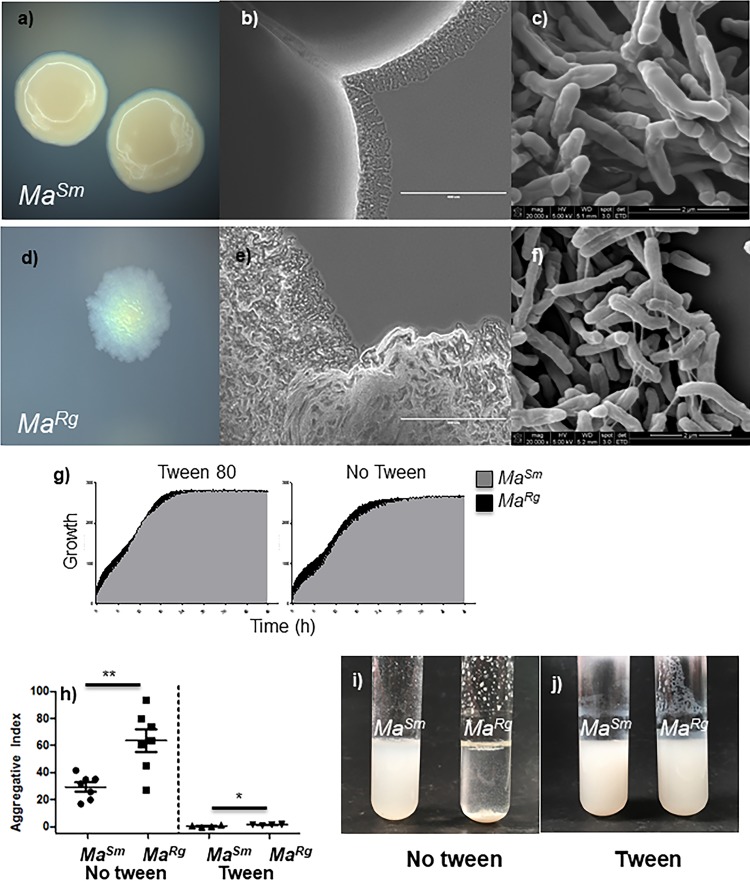
Characterization of M. abscessus smooth (*Ma^Sm^*) and rough (*Ma^Rg^*) variant isolates. (a and d) *Ma^Sm^* and *Ma^Rg^* isolates were distinguishable by colony morphotype on 7H10 agar. (b, c, e, and f) *Ma^Rg^* colonies were also distinguishable by cording at the peripheries of colonies on agar at low magnification (b and e), but not by SEM (c and f). (g) Isolated *Ma^Rg^* and *Ma^Sm^* colonies were grown in 7H9 broth. Growth was similar for the two variants in 7H9 broth with or without Tween 80. (h) *Ma^Rg^* was more aggregative than *Ma^Sm^*. (i) When cultures were removed from shaking after 15 min, *Ma^Sm^* remained suspended, but *Ma^Rg^* rapidly settled out in the absence of Tween. (j) Aggregation was significantly reduced with 0.5% Tween.

M. abscessus smooth and rough variants have been shown to differ in sliding motility and the ability to colonize the surfaces of pegs in MBEC plates (32). We found that *Ma^Rg^* settled rapidly when not shaken, and we hypothesized that this might be due to its ability to aggregate. *Ma^Sm^* and *Ma^Rg^* isolates were readily distinguishable by colony morphology on 7H10 agar (Fig. 1a and d). By use of a low-magnification stereomicroscope, *Ma^Rg^* colonies were also distinguished by cording at the edge of the colonies (Fig. 1b and e). Colony variant cell structures were not distinguishable by scanning electron microscopy (SEM) (Fig. 1c and f), although interconnecting threadlike structures are visible on rough bacilli (Fig. f). Growth in 7H9 broth with and without Tween 80 (OmniLog) was similar for the two variants (Fig. 1g). The optical density at 600 nm (OD_600_) of isolated *Ma^Rg^* or *Ma^Sm^* grown with shaking for 48 h with or without Tween showed that *Ma^Rg^* settled within 15 min in the absence of Tween, whereas *Ma^Sm^* remained suspended during this time, indicating that *Ma^Rg^* was significantly more aggregative than *Ma^Sm^* (Fig. 1h). *Ma^Rg^* but not *Ma^Sm^* aggregates were visible on the side and bottom of the test tube, and aggregation was significantly inhibited in the presence of Tween (Fig. 1i and j).

### *Ma^Rg^* and *Ma^Sm^* form biofilms with distinct phenotypes.

**FIG 2:**
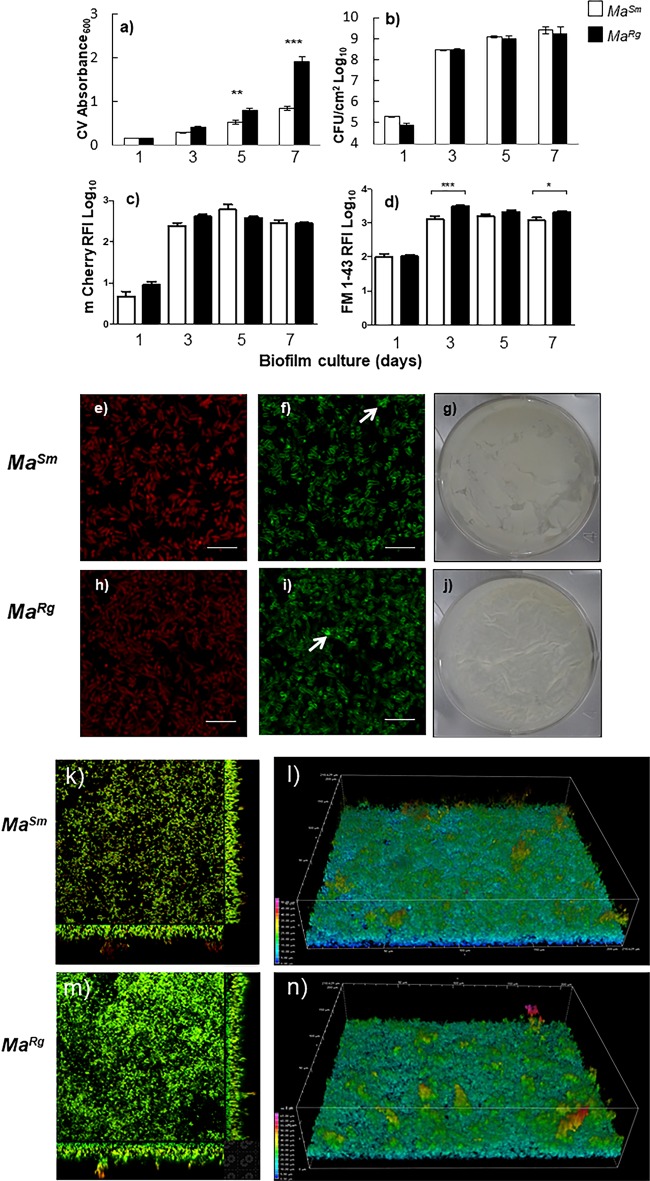
*Ma^Sm^* and *Ma^Rg^* variants each develop aggregated biofilm structures over time. (a and d) Biomass (expressed as CV absorbance) was greater with *Ma^Rg^* (filled bars) than with *Ma^Sm^* (open bars) (a), and similar results were obtained using the lipophilic probe FM 1-43 to label variants (d). (b and c) Biofilm development did not differ statistically (*P* > 0.05) between variants when measured by CFU per square centimeter (b) or by mCherry relative fluorescence intensity (RFI) (c). Error bars, standard errors of the means. CFU data represent 3 replicate wells and 3 biological replicates (*n* = 9); CV and RFI data represent 6 replicate wells and 3 biological replicates (*n* = 18). (g and j) Pellicle biofilms showed distinct morphologies for *Ma^Rg^* and *Ma^Sm^* variants after 7 days. (e and h) Confocal slices showed levels of mCherry-expressing *Ma^Sm^* (e) and *Ma^Rg^* (h) to be similar. (f and i) The lipophilic probe FM 1-43 showed higher RFI for *Ma^Rg^* (i) than for *Ma^Sm^* (f) (arrows indicate extracellular lipid). (k through n) Finally, orthogonal confocal z-stack images (k and m) and 3-D images pseudocolored to highlight the depth of bacterial biofilms (l and n) showed that complex aggregated biofilm structures were present after 48 h for both variants.

Since *Ma^Rg^* was more aggregative than *Ma^Sm^*, we hypothesized that this would affect its biofilm-forming capacity. Due to the lack of a consensus definition of biofilms, particularly with respect to mycobacteria, we used several assays to measure biofilm formation with isolated *Ma^Rg^* or *Ma^Sm^* variants over 7 days. Crystal violet (CV) absorbance, a determination of biomass that includes the nonspecific measurement of extracellular matrix material, was 2-fold greater with *Ma^Rg^* after day 3 (*P* ≤ 0.001) (Fig. 2a). Enumeration of CFU per square centimeter, however, indicated that the numbers of bacteria in the biofilms of the two variants did not differ statistically (*P* > 0.05) (Fig. 2b). We also quantified biofilm formation by measuring the relative fluorescence intensity (RFI) of mCherry-expressing *Ma^Sm^* or *Ma^Rg^* over time. In agreement with the data on CFU per square centimeter, mCherry RFI showed no significant differences between the two variants (Fig. 2c, e, and h). Each morphotype also exhibited a higher RFI when probed with lipophilic FM 1-43 over time. However, *Ma^Rg^* showed significantly more lipophilic material associated with biofilm aggregates than did *Ma^Sm^* at days 3 and 7 (Fig. 2d, f, and i). After 3 days, biofilms were visible on the surface as a pellicle and on the bottoms of wells; at day 7, the *Ma^Sm^* pellicle appeared oleaginous and the *Ma^Rg^* pellicle waxy (Fig. 2g and j). Neither variant exhibited robust attachment to surfaces, even though large, structurally complex bacterial aggregates were visible in wells with both variants (Fig. 2k and m). *Ma^Sm^* or *Ma^Rg^* aggregates exhibited lipophilic-rich structures (Fig. 2l and n) generally colocalized with mCherry, with some evidence of extracellular lipid in aggregates. Thus, *Ma^Sm^* and *Ma^Rg^* biofilms each demonstrated 3-dimensional (3-D) biofilm-like aggregates that were structurally contiguous.

### *Ma^Sm^* and *Ma^Rg^* biofilm formation confers tolerance to antimicrobial treatment.

**FIG 3:**
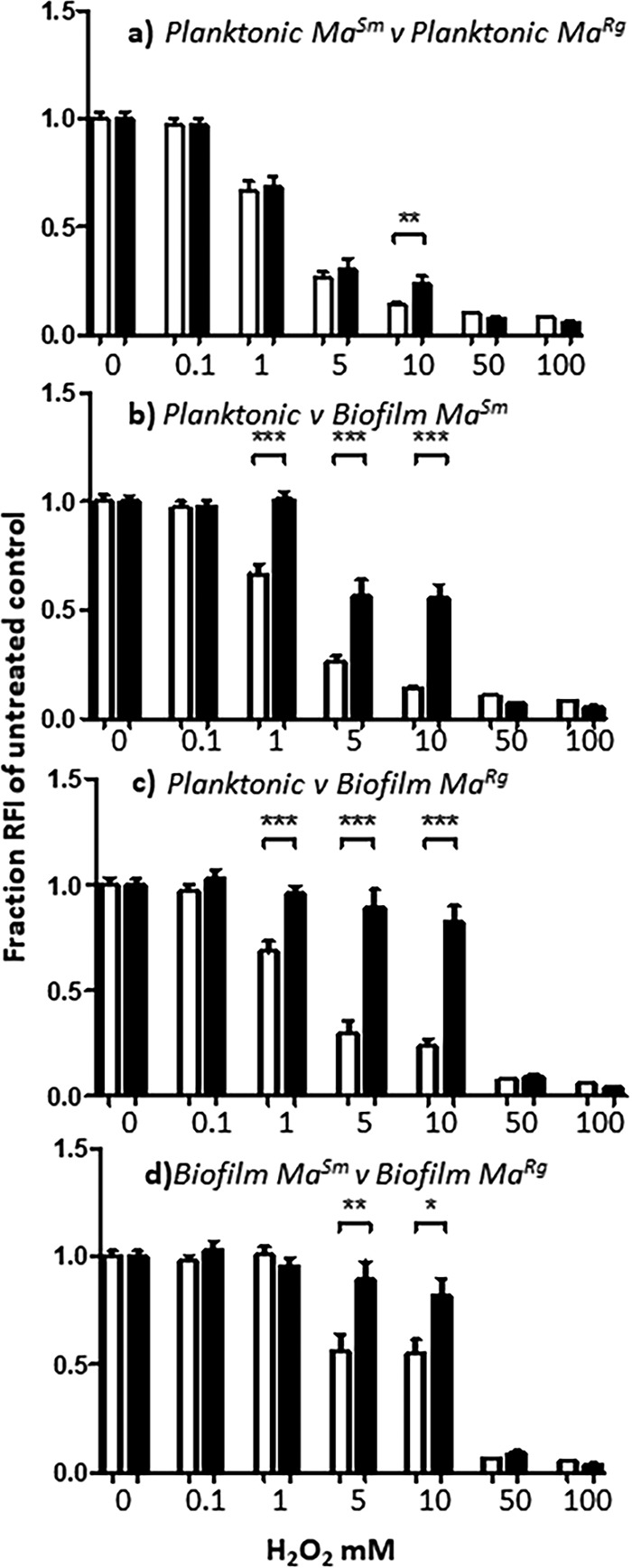
Biofilm *Ma^Sm^* or *Ma^Rg^* is more tolerant of hydrogen peroxide than planktonic variants. (a) Planktonic *Ma^Sm^* or *Ma^Rg^* was susceptible to concentrations of H_2_O_2_ at or above 1 mM, and *Ma^Sm^* was more susceptible to 10 mM H_2_O_2_ than *Ma^Rg^*. (b and c) *Ma^Sm^* or *Ma^Rg^* biofilms were significantly more tolerant of H_2_O_2_ at 1 to 10 mM concentrations than planktonic *Ma^Sm^* or *Ma^Rg^*, respectively. (d) *Ma^Rg^* biofilms were more tolerant of H_2_O_2_ at concentrations between 5 and 10 mM than *Ma^Sm^* biofilms. Data represent 6 wells per experiment, with 3 biological replicates (*n* = 18). *, *P* < 0.05; **, *P* < 0.01; ***, *P* < 0.001.

Antimicrobial tolerance is a characteristic criterion for microbial biofilms (26, 43). To further interrogate whether *Ma^Rg^* exhibited biofilm-like behavior, we tested the ability of each variant to withstand antimicrobial treatment when grown planktonically or as biofilms. Virulent mycobacteria can resist host defense strategies, and therefore, we examined the susceptibilities of the *Ma^Sm^* and *Ma^Rg^* variants to hydrogen peroxide (H_2_O_2_) or low pH (47, 48). Planktonic cells showed a reduction in survival—indicated by the reduced RFI of mCherry transformed cells—at H_2_O_2_ concentrations of 1 mM and above (Fig. 3a); however, *Ma^Sm^* and *Ma^Rg^* biofilm-like aggregates were each more resistant to H_2_O_2_ between 1 and 10 mM (Fig. 3b and c) (*P*, <0.001 by an unpaired *t* test).

**FIG 4:**
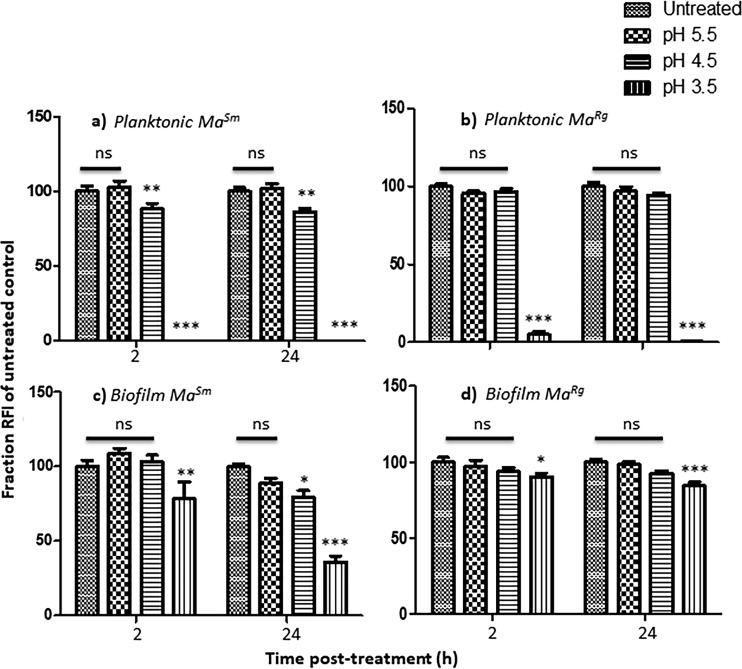
Biofilm *Ma^Sm^* or *Ma^Rg^* is more tolerant of low pH than planktonic variants. (a and b) At pH 5.5, the RFIs of planktonic *Ma^Sm^* and *Ma^Rg^* were not significantly different from the RFIs of untreated bacteria. *Ma^Sm^*, but not *Ma^Rg^*, showed a significant difference at pH 4.5. Both showed significant differences at pH 3.5. (c and d) In contrast, *Ma^Sm^* and *Ma^Rg^* showed no statistical difference between untreated biofilms and those treated at pH 4.5 for 2 or 24 h. *Ma^Sm^* and *Ma^Rg^* biofilms treated at pH 3.5 were significantly different from those under all other conditions by two-way ANOVA and were significantly different from each other by a *t* test (*P* < 0.001). Data represent 2 experiments with 6 wells per experiment. ns, not significant (*P* > 0.05); *, *P* < 0.05; **, *P* < 0.01; ***, *P* < 0.001.

Planktonic *Ma^Sm^* was unaffected at pH 5.5, with no significant reduction in RFI after 2 h from that of untreated controls (*P* > 0.05), but its RFI was significantly reduced at pH 4.5 (*P* < 0.01) (Fig. 4a). *Ma^Rg^*, however, tolerated pH 4.5 (*P* > 0.05). Both variants were susceptible to pH 3.5 (*P* < 0.001). After 2 h at pH 4.5, biofilm *Ma^Sm^* showed no difference in RFI from untreated *Ma^Sm^* or *Ma^Sm^* at pH 5.5 (*P* > 0.05). However, there was a significant difference between variants at pH 3.5 (*P*, <0.001 by *t* test) at 24 h, evidenced also by a 2- to 3-log reduction in *Ma^Sm^* of bacterial CFUs compared to untreated controls, but only a ∼1-log reduction in *Ma^Rg^*, indicating that biofilm aggregates were significantly more tolerant of low pH and that *Ma^Rg^* was more tolerant than *Ma^Sm^* (Fig. 4c and d; Table 1).

**TABLE 1:** CFU reduction

Antimicrobial agent^*a*^	Log CFU reduction^*b*^ under the following growth condition:			
	Planktonic		Biofilm	
	*Ma*^*Sm*^	*Ma*^*Rg*^	*Ma*^*Sm*^	*Ma*^*Rg*^
H_2_O_2_ (mM)				
1	<1	<1	<1	<1
5	<1	<1	<1	<1
10	1.9	1.2	<1	<1
50	>7*	>7*	4.8	2.3
100	>7*	>7*	>7*	6.2
HCl (pH)				
5.5	ND	ND	<1	<1
4.5	ND	ND	<1	<1
3.5	4.9	3.4	2.5	1.1
Amikacin (256 μg/ml)	ND	ND	<1	<1
Azithromycin (256 μg/ml)	ND	ND	0	0
Acetic acid (%)				
2-h exposure				
1	5.3	4	1.3	1.3
2.5	>7*	>7*	>7*	>7*
5	>7*	>7*	>7*	>7*
30-min exposure				
1	1	<1	<1	<1
2.5	4	2	2.2	2.9
5	>7*	>7*	>7*	>7*

a For all agents or stresses except acetic acid, the exposure time was 24 h.

b ND, no data; *, detection limit.

**FIG 5:**
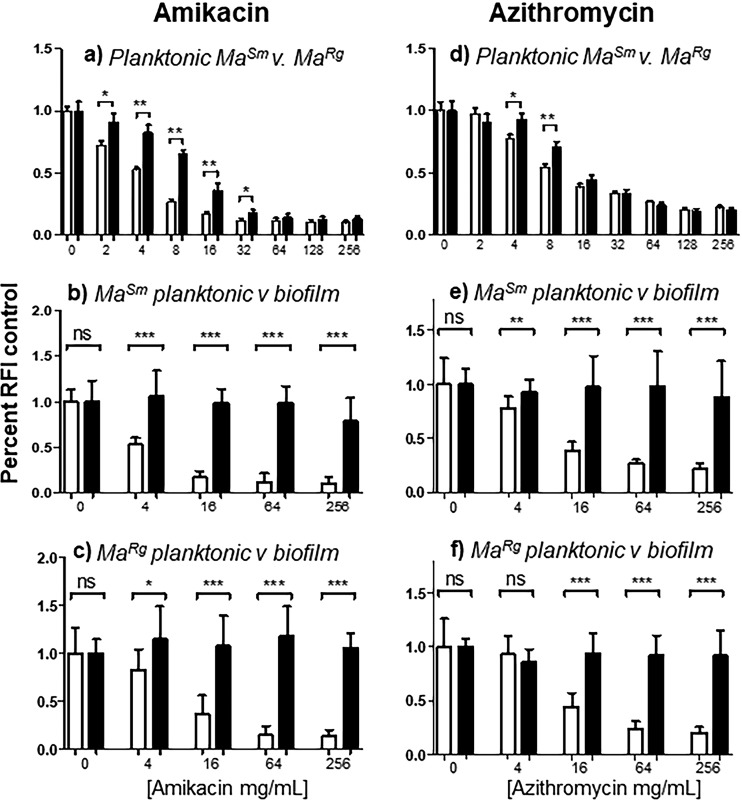
Biofilm *Ma^Sm^* or *Ma^Rg^* is more refractory to antibiotic treatment than planktonic variants. (a) Planktonic *Ma^Sm^* showed a significantly lower mCherry RFI than planktonic *Ma^Rg^* in response to amikacin concentrations between 2 and 32 μg/ml. (d) Planktonic *Ma^Sm^* also showed a significantly lower RFI than planktonic *Ma^Rg^* in response to azithromycin concentrations of 4 to 8 μg/ml. (b, c, e, and f) However, biofilms of both M. abscessus variants were significantly more tolerant of antibiotic treatment than planktonic bacteria. (b and c) Concentrations of amikacin that resulted in reduced RFIs for planktonic cells failed to result in significant reductions in the RFIs of biofilms of either variant. (e and f) A similar effect was seen with azithromycin. Data represent 6 wells for each of 2 biological replicates (*n* = 12). *, *P* < 0.05; **, *P* < 0.01; ***, *P* < 0.001.

Biofilm formation also results in antibiotic tolerance greater than that of planktonic cells (26, 43). Amikacin treatment at concentrations above 2 μg/ml resulted in a significantly lower RFI for planktonic *Ma^Sm^*, and a similar result was observed with azithromycin treatment above concentrations of 4 μg/ml (Fig. 5a and d). The *Ma^Rg^* RFI was also reduced with each antibiotic; however, the reduction was significantly less than for planktonic *Ma^Sm^* at amikacin concentrations between 2 and 32 μg/ml and at azithromycin concentrations between 4 and 8 μg/ml. These results are in broad agreement with the MICs reported for amikacin (2 to 4 μg/ml) and azithromycin (8 to 16 μg/ml) but suggest that *Ma^Rg^* is more recalcitrant to antibiotic treatment than *Ma^Sm^*. The results are also consistent with data obtained with each variant on 7H10 agar showing that *Ma^Sm^* was more susceptible to lower concentrations of amikacin or azithromycin than *Ma^Rg^* by zones of inhibition (data not shown).

In contrast to the results with planktonic bacteria, the RFIs of *Ma^Sm^* or *Ma^Rg^* biofilms treated with high concentrations of amikacin or azithromycin were unaffected after 24 h, as seen by comparison to untreated controls, providing evidence that *Ma^Sm^* and *Ma^Rg^* biofilm aggregates exhibited tolerance to each of these antibiotics (Fig. 5b, c, e, and f). Although planktonic *Ma^Sm^* and *Ma^Rg^* showed significant reductions in RFI with significant differences between variants, biofilm-like aggregates of both variants were tolerant to all concentrations of antibiotic after 24 h of treatment. Extended contact times of 48 and 72 h showed no reductions in RFI for biofilm bacteria (data not shown). By all criteria used to distinguish biofilms, including antimicrobial tolerance, M. abscessus colony morphology variants were comparable.

### *Ma^Rg^* survives significantly better than *Ma^Sm^* in untreated or azithromycin-treated differentiated THP-1 macrophages despite similar uptake, but variants survive equally well in macrophages treated with amikacin.

**FIG 6:**
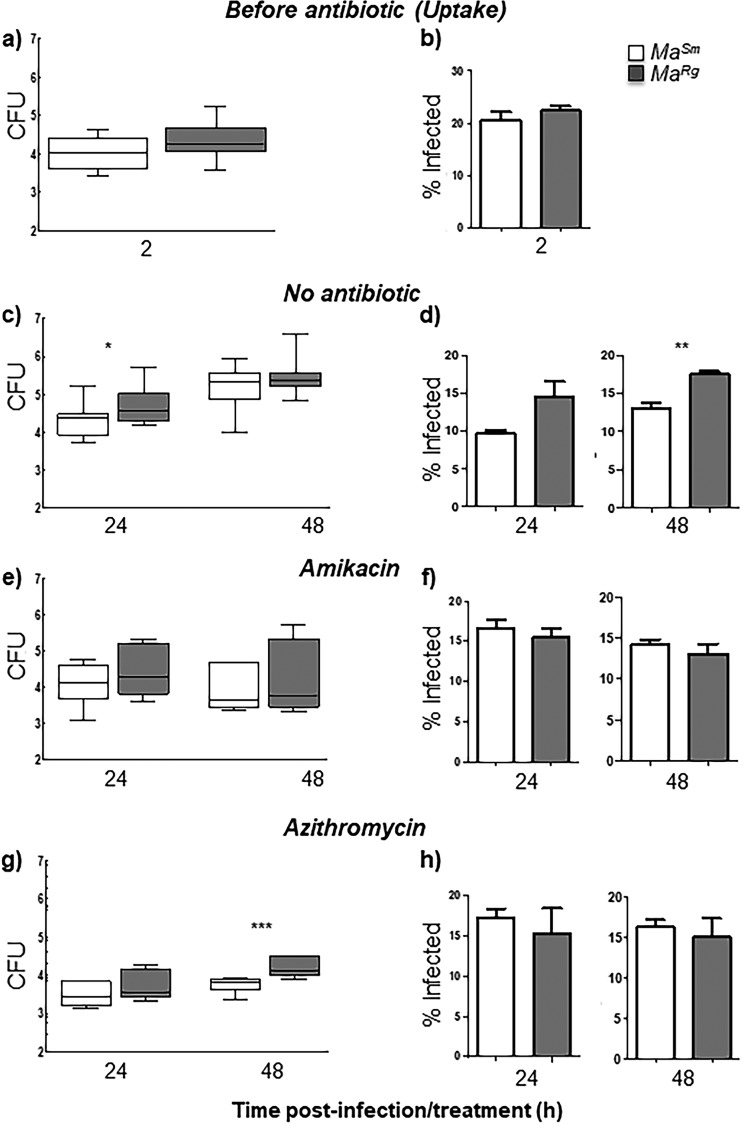
*Ma^Sm^* or *Ma^Rg^* survives inside THP-1 cells with or without antibiotic treatment. (a and b) Uptake by THP-1 cells infected with opsonized *Ma^Sm^* (open bars) or *Ma^Rg^* (shaded bars) at an MOI of 2.5 for 2 h did not differ significantly between variants as determined by CFU (a) or by microscopy (b). (c to h) Infected-cell monolayers treated with antibiotics had similar intracellular burdens to cells without antibiotic treatment at 48 h. (e and f) Infected-cell monolayers treated with amikacin show that both *Ma^Sm^* and *Ma^Rg^* survive intracellularly in macrophage-like THP-1 cells over 48 h. (g and h) Azithromycin reduced the number of intracellular bacteria; however, *Ma^Rg^* was less susceptible to azithromycin at 48 h. For CFU experiments, data represent 3 biological replicates (5 replicates for no-antibiotic controls) with triplicate wells per experiment. For microscopic analysis, data represent 2 biological replicates (3 for azithromycin) with duplicate plates per experiment. (*, *P* < 0.05; **, *P* < 0.01; ***, *P* < 0.001).

Since M. abscessus variants have been shown to differ in their invasiveness in cells (32), we studied *Ma^Sm^* and *Ma^Rg^* uptake in phorbol 12-myristate 13-acetate (PMA)-differentiated human THP-1 cells and tested whether antibiotic treatment differentially affects intracellular survival. At a multiplicity of infection (MOI) of 2.5, there was no difference in intracellular *Ma^Sm^* or *Ma^Rg^* levels after 2 h of infection as determined by CFU or by confocal microscopy, indicating similar uptake kinetics by THP-1 cells of each variant (Fig. 6a and b). However, after 24 h without antibiotic treatment, there was significantly more *Ma^Rg^* than *Ma^Sm^* in THP-1 cells by CFU counts (*P* < 0.05) (Fig. 6c). This difference was not seen at 48 h by CFU counts (Fig. 6c) but was seen at this time point by microscopy (*P* < 0.01) (Fig. 6d). Amikacin treatment inhibited the intracellular growth of *Ma^Rg^* or *Ma^Sm^* relative to that in THP-1 cells without antibiotic and showed no significant difference between variants over 48 h of treatment, a finding commensurate with the percentages of infected cells observed using confocal microscopy (Fig. 6e and f). Azithromycin effectively reduced intracellular *Ma^Sm^* or *Ma^Rg^* levels from those with no antibiotic treatment; however, by 48 h, there was significantly more intracellular *Ma^Rg^* than *Ma^Sm^* (*P* < 0.001) (Fig. 6g). When macrophages were examined microscopically, approximately 10 to 20% of cells were infected at 2 h, and this percentage remained consistent over the infection period for both variants (Fig. 6b, d, f, and h; see also Fig. S1 in the supplemental material). These data demonstrate that whereas both variants can survive in macrophages, *Ma^Rg^* may have a survival advantage.

### Acetic acid rapidly kills *Ma^Rg^* and *Ma^Sm^* biofilms.

**FIG 7:**
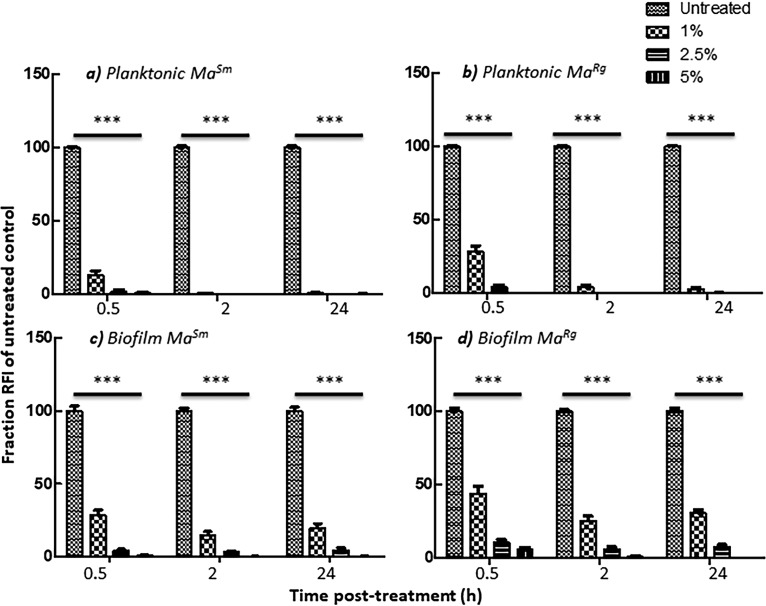
Biofilm *Ma^Sm^* or *Ma^Rg^* is susceptible to acetic acid. (a and b) Planktonic *Ma^Sm^* or *Ma^Rg^* treated with acetic acid concentrations of 1% or higher was statistically different from untreated *Ma^Sm^* or *Ma^Rg^* by 30 min posttreatment. (c and d) Significantly, this was also observed for *Ma^Sm^* and *Ma^Rg^* biofilms with 2.5% or 5% acetic acid after only 30 min. There was no significant difference between *Ma^Sm^* and *Ma^Rg^* by *t* test (*P* > 0.05). Data represent 5 biological replicates with 6 wells per experiment. ***, *P* < 0.001.

Acetic acid is an effective tuberculocidal disinfectant that is also effective against M. abscessus, although distinct morphotypes or biofilms have not been evaluated previously (49). We therefore tested acetic acid against highly tolerant M. abscessus variant biofilms. Planktonic *Ma^Sm^* or *Ma^Rg^* treated with acetic acid was significantly different from untreated *Ma^Sm^* or *Ma^Rg^* (*P* < 0.001) at all concentrations and time points, indicating susceptibility (Fig. 7a and b). Treatment with 1% acetic acid resulted in a reduction of more than 5 log units for planktonic *Ma^Sm^* and 4 log units for planktonic *Ma^Rg^* at 2 h (Table 1). The RFIs of *Ma^Sm^* or *Ma^Rg^* biofilms were also significantly reduced in a dose-dependent manner after only 30 min (*P* < 0.001), although 1% acetic acid resulted in only a 1-log reduction for biofilms of each variant (Table 1). Notably, levels of both planktonic and biofilm *Ma^Sm^* and *Ma^Rg^* were reduced to the detection limits, as determined by RFI and CFU counts, with exposure to 2.5% acetic acid for 2 h (Fig. 7a to d; Table 1). Remarkably, after only 30 min of exposure to 5% acetic acid, the RFIs of both planktonic variants were reduced to the detection limits (Fig. 7a to d), with a >7 log reduction in CFU counts (Table 1).

## DISCUSSION

Despite the association of *Ma^Rg^* with increased pathogenicity (10, 36), few studies have compared M. abscessus colony variants for pathogenic properties. Such studies have suggested that biofilm formation is restricted to the *Ma^Sm^* variant (32, 33, 40). In contrast, using isolated isogenic colony morphology variants from the sequenced M. abscessus 19977^T^ reference strain, we found that *Ma^Rg^* is more aggregative than *Ma^Sm^* and that each variant forms biofilms with distinct phenotypes over 7 days. *Ma^Sm^* and *Ma^Rg^* have similar numbers of bacteria (in CFU counts per square centimeter) in biofilms, a finding consistent with mCherry *Ma^Sm^* or *Ma^Rg^* biofilm RFIs over time. However, *Ma^Rg^* showed significantly more biofilm biomass by CV OD_600_ than *Ma^Sm^*, as well as increased RFI with the lipophilic probe FM 1-43, possibly due to more extracellular lipid.

*Ma^Sm^* and *Ma^Rg^* variants, including those of strain 19977, differ in the expression of GPLs (32, 33, 35, 37, 50, 51). GPLs are associated with sliding motility and play a role in the development of biofilms in both nonpathogenic and pathogenic mycobacteria (32, 34, 52, 53). Our results indicate that despite low GPL expression, *Ma^Rg^* still forms pellicles and exhibits a phenotype that results in the accumulation of multicellular biofilm structures and biofilm-like aggregates with small foci of extracellular lipid, not previously shown for a rough M. abscessus variant. It is unclear how GPL expression affects aggregation by each variant. The deletion of *mmpL4b*, a gene in the GPL biosynthetic pathway, was found to abrogate the ability of M. abscessus to colonize surfaces and to enhance its ability to replicate in human macrophages (34). The MmpL family of proteins plays a role in the biosynthesis of the cell envelope, and the mutation resulted in defective GPL production and/or transport in the rough variant and the capacity to produce cords *in vitro* (50). Rough variants of M. abscessus and Mycobacterium bolletii exhibit cording morphology in a zebrafish model of infection, and genetic analysis of a spontaneous rough variant of *M. bolletii* recently identified isogenic smooth and rough variants that differed by a single mutation in *mmpL4a* (37, 50). We saw no evidence of cording in *Ma^Sm^* aggregates; however, we observed variable cording morphology in *Ma^Rg^* aggregates, suggesting that cording occurs under specific growth conditions.

Motility *per se* is not required for biofilm development in many bacteria, although aggregation is necessary (26, 42, 44, 45). The aggregative phenotype exhibited by *Ma^Rg^* may result in an antibiotic-tolerant phenotype similar to that of rugose-colony variants of P. aeruginosa from CF patients (25). Notably the M. abscessus genome possesses nonmycobacterial virulence genes, including some from P. aeruginosa (46). Our results suggest that neither biofilm development, nor survival in macrophages is morphotype restricted and that patients may be colonized with either or both M. abscessus variants. The expression of different lipids on the cell wall, however, may lead to variant-specific host cell responses that are important in delineating mechanisms that contribute to persistent infection and to M. abscessus virulence (41).

Our studies differ from previous studies that showed that *Ma^Rg^* failed to form biofilms. First, those studies assessed biofilm formation using a method based on bacterial attachment to pegs in MBEC microtiter plates. That method, however, has limitations in measuring bacterial aggregates (such as those observed with *Ma^Rg^*), which would be more likely to settle on the well bottom than to attach to the pegs (54). Second, previous studies measured biofilm formation up to 72 h, whereas *Ma^Rg^* pellicle formation took longer in our study, although the antimicrobial-tolerant phenotype was present after 24 h. Third, previous studies used a clinical isolate characterized by a spontaneous rough-to-smooth reversion, and that strain may exhibit a strain-specific nonbiofilm phenotype, in contrast to M. abscessus 19977.

Antimicrobial tolerance is a hallmark of biofilm formation (26, 43). Our data show that M. abscessus biofilm-like aggregates of each variant are significantly more tolerant of acidic pHs than planktonic bacteria. Biofilm aggregates also conferred significantly better survival than that of planktonic cells when bacteria were treated with H_2_O_2_, but biofilms were significantly inhibited with 100 mM H_2_O_2_. M. abscessus biofilms were also significantly more tolerant of two antibiotics commonly used to treat mycobacterial infection: amikacin and azithromycin. When planktonic *Ma^Sm^* or *Ma^Rg^* was treated with amikacin or azithromycin, its level decreased significantly, in a dose-dependent manner, from that of untreated controls. In this case, *Ma^Rg^* was more recalcitrant to antibiotic treatment at concentrations between 2 and 32 μg/ml of amikacin and between 4 and 8 μg/ml of azithromycin. When *Ma^Sm^* and *Ma^Rg^* formed biofilm aggregates, however, each tolerated an antibiotic concentration of 256 μg/ml with no significant reduction in RFI; this concentration is 10 to 100 times the MIC, which is higher than the minimal bactericidal concentration (MBC)/MIC ratio (>4) generally accepted for bactericidal or bacteriostatic antibiotics (12). Thus, by all criteria used for biofilms, including antimicrobial tolerance, M. abscessus colony morphology variants were comparable. Overall our data suggest that *in vivo* conditions where M. abscessus may aggregate can contribute to recalcitrance to antibiotic treatment. Furthermore, these results may help to explain the high rates of ototoxicity associated with the high concentration of amikacin required to achieve maximum killing in 70% of patients (55).

The ability to survive intracellularly in macrophages can also contribute to mycobacterial persistence, and it has been suggested that the *Ma^Sm^* variant is less invasive (32–34). Our results show that the levels of uptake of opsonized *Ma^Sm^* or *Ma^Rg^* by THP-1 macrophages are similar. However, THP-1 macrophages infected with *Ma^Sm^* or *Ma^Rg^* without antibiotic treatment harbored significantly more *Ma^Rg^* than *Ma^Sm^* at 24 h, suggesting that while *Ma^Rg^* may not be more invasive, it may survive better intracellularly. Alternatively, *Ma^Rg^* may be able to replicate better than *Ma^Sm^* in human macrophages (34). We are currently investigating colony variant survival in human monocyte-derived macrophages in order to better assess these possibilities. In contrast to the results obtained without antibiotics, survival in macrophages treated with amikacin did not differ between the variants at 48 h, suggesting a bacteriostatic effect on both variants or diminished antibiotic levels in THP-1 macrophages. Amikacin is widely used to treat M. abscessus infections, and a recent study showed that M. abscessus clinical strains exhibited bacteriostatic responses to amikacin, suggesting that the discrepancy between antibiotic susceptibilities *in vitro* and *in vivo* may be compounded by variable drug concentrations under *in vivo* conditions (12).

More *Ma^Rg^* than *Ma^Sm^* was present after 48 h of treatment with azithromycin, suggesting that although each variant can survive intracellularly, *Ma^Rg^* may be more recalcitrant to this antibiotic than *Ma^Sm^*, in agreement with our data showing that this variant is more refractory than *Ma^Sm^* to other antimicrobial treatments. Azithromycin penetrates cells more effectively than amikacin, in agreement with the reduced intracellular burden of *Ma^Sm^* or *Ma^Rg^* in THP-1 macrophages and with a study showing that M. abscessus survived intracellularly in human macrophages treated with clarithromycin (40). Although our studies with mCherry-labeled planktonic M. abscessus variants showed a range of antibiotic concentrations similar to published results, microscopic data with both mCherry-labeled variants and CFU data with nontransformed variants indicate that intracellular *Ma^Sm^* and *Ma^Rg^* both persisted over 48 h. Azithromycin has multiple effects on host cell responses, including increasing ion efflux, reducing tumor necrosis factor alpha (TNF-α) production, and interfering with autophagy, and it is possible that these effects contributed to the survival of M. abscessus despite the ability of azithromycin to concentrate inside cells (56).

Collectively, our results indicate that the antibiotic concentrations required to kill either M. abscessus variant in biofilm-like aggregates or in host cells may be clinically unachievable or may result in cytotoxic adverse effects because of high, prolonged drug concentrations. Reports of experiments with immunodeficient mice show that although amikacin or azithromycin treatment resulted in lower bacterial burdens *in vivo*, antibiotic treatment did not eradicate M. abscessus in the lungs (19, 57). In a study of patients with pulmonary infection, combination therapy with amikacin, cefoxitin, and clarithromycin failed to successfully treat pulmonary M. abscessus (13). Our study further highlights the limited efficacy of antibiotic therapy in treating M. abscessus infections and emphasizes the need for new therapeutic approaches.

Historically, acquisition of M. abscessus has been attributed to the exposure of individuals to environmental sources. Recent studies, however, report that in some outbreaks, M. abscessus strains in CF patients have higher genomic identity than would be predicted by acquisition from environmental sources, suggesting that some clones may be transmitted from nosocomial sources (24). M. abscessus aggregates harboring viable bacteria that were refractory to antimicrobial treatment were recently demonstrated in a resected lung cavity in a patient with COPD (31). Biofilm M. abscessus was also found embedded in the alveolar walls of an end-stage explanted lung, and in mucus in the conductive zone of the airways, in a patient with CF (28). In both cases, infectious aerosols containing aggregated bacilli may facilitate M. abscessus transmission. M. abscessus CF isolates survived in aerosolized droplet nucleus particles (58), and particulates enhanced fomite survival (59). Biofilm formation by Streptococcus pneumoniae, a pathogen thought to die quickly outside the human host, resulted in prolonged survival on fomites (60). Together, these studies suggest that biofilm-like aggregates may survive outside the host and contribute to the nonrandom global transmission of M. abscessus clones in CF patients (24). We propose that the ability of M. abscessus variants to form biofilms should be recognized as a virulence factor.

Although biofilm formation by *Ma^Rg^* and *Ma^Sm^* significantly increases bacterial survival of antimicrobial treatment, acetic acid was remarkably effective at killing M. abscessus. Acetic acid can kill several species of planktonic mycobacteria with short contact times (49). Our data show that 5% acetic acid kills planktonic as well as biofilm *Ma^Rg^* and *Ma^Sm^* within 30 min, and they extend the existing evidence that acetic acid effectively eradicates M. abscessus to include biofilms. Thus, acetic acid may help to prevent transmission in clinical settings where aggregates of M. abscessus may be present on fomites.

## MATERIALS AND METHODS

### Bacteria.

M. abscessus ATCC 19977 was obtained from the American Type Culture Collection (Manassas, VA), reconstituted, and grown as directed for the establishment of frozen stocks. Since M. abscessus contains a mixture of both smooth and rough morphotypic variants, frozen stocks were streaked to isolation and individual variants cultured on 7H10 agar containing 10% oleic acid-albumin-dextrose-catalase (OADC) and glycerol. For frozen stocks, each morphotype was scraped into 7H9 broth containing 10% OADC–20% glycerol and was frozen at −80°C. For mCherry transformation, each morphotype was grown and rinsed; an mCherry cassette with kanamycin resistance (provided by Sarah Fortune, Harvard University) was electroporated into M. abscessus; and transformants were selected on kanamycin-containing selective agar medium. Electrocompetent 19977 cells were prepared by growing bacterial cultures to mid-log phase (OD_600_, 1.0) and harvesting the bacterial cells by centrifugation at 4°C and 2,000 × *g* for 10 min. Cells were washed three times in freshly prepared sterile-filtered, ice-cold 10% glycerol–H_2_O, and pellets were then resuspended in 1 ml ice-cold 10% glycerol–H_2_O. Electroporation of mCherry plasmid construct DNA was carried out using a Bio-Rad Gene Pulser II system (Bio-Rad, Hercules, CA) set at 2,500 V, 1,000 Ω, and 25 μF, according to the manufacturer's instructions. Briefly, 0.5 to 1.0 μg of DNA was added to 200 μl of washed electrocompetent cells, mixed gently in an electroporation cuvette, incubated on ice, and inserted into an electroporation chamber, followed by an electrical pulse. Transformants were grown on 7H10 agar containing 100 μg/ml kanamycin for 3 to 5 days, and frozen stocks were prepared as described above. Bacteria were grown from frozen individual colony variant stocks on 7H10 plates for 5 to 7 days at 37°C under 5% CO_2_. Morphotypes were checked using a stereoscopic microscope (Stereo Zoom microscope; AmScope, USA) and were plated separately for use in experiments.

### Planktonic and biofilm growth.

Single cells of each M. abscessus morphotype were obtained by direct counts as described previously (61). Briefly, for each experiment, isolated morphotypic colony variants were placed in Eppendorf tubes, each containing 1 ml of 7H9 broth–OADC–0.5% Tween 80, pulse vortexed with glass beads, and allowed to settle for 30 min, after which 600 μl of the supernatant was transferred to a second tube. After 10 min, 300 μl was transferred from each tube to a new tube. Bacteria were transferred to a Petroff-Hausser chamber and were counted. For CFU experiments, 4 × 10^8^ bacteria in 4 ml of 7H9 broth–OADC without Tween were dispensed into 6-well tissue culture plates (Costar), and half the medium was replenished daily. For the assessment of biomass using crystal violet (CV) or for the determination of fluorescence intensity, 100 μl (1 × 10^7^ bacteria) was dispensed into 96-well plates (Costar) as described elsewhere (62). Cultures were incubated at 37°C under 5% CO_2_, and biofilm development was measured using a SpectraMax plate reader (Molecular Devices, Sunnyvale, CA) over 7 days.

Biofilm development was also determined using mCherry-transformed bacteria. *Ma^Sm^* and *Ma^Rg^* variants were each inoculated into 96-well black, clear-bottom plates (Costar) at a density of 1 × 10^7^ bacteria/well. At each time point, wells were rinsed to remove suspended bacteria, and mCherry (red fluorescence) (excitation wavelength, 575 nm; emission wavelength, 610 nm) was quantified using a SpectraMax plate reader to determine relative fluorescence intensity (RFI). For the measurement of lipid, biofilms in separate wells were rinsed, incubated with FM 1-43 (Molecular Probes, Eugene, OR) as per the manufacturer's instructions, and rinsed before RFI measurement.

### Bacterial growth and aggregative assays.

The growth of each variant was determined using the OmniLog PhenoType MicroArray system (Biolog Inc., Hayward, CA). *Ma^Sm^* or *Ma^Rg^* was grown for as long as 48 h in the OmniLog incubator in 7H9 broth with OADC, with or without Tween 80, and in the presence of Biolog Redox Dye A at a density of 1 × 10^7^ bacteria/well. Readings were taken every 15 min.

The ability of M. abscessus to aggregate was assessed using an optical density aggregative index as described elsewhere (63) with modifications. Briefly, *Ma^Sm^* or *Ma^Rg^* was grown for 48 h in 7H9 broth with OADC, with or without Tween 80. After removal from the shaking incubator, cultures were gently agitated, and the OD_600_ was taken at time 1 (*T*_1_), and again after 15 min (*T*_2_), for each variant. The aggregative index (AI) was calculated as OD_*T*1_ − OD_*T*2_/OD_*T*1_ × 100.

### Antimicrobial/antibiotic assays.

Amikacin (OSUMC Pharmacy - main) and azithromycin (Sigma-Aldrich) were added to RPMI medium with 10% heat-inactivated fetal bovine serum (HI-FBS) and were diluted to obtain a range of final concentrations between 2 μg/ml and 256 μg/ml. Each antibiotic was added to planktonic suspensions containing 1 × 10^7^ mCherry-transformed *Ma^Sm^* or *Ma^Rg^* bacteria/well in 96-well black, clear-bottom plates (Costar), and the mixture was incubated at 37°C under 5% CO_2_ for 24 to 48 h. Inhibitory activity was assessed by measuring RFI using a modification of the 96-well assay. The MIC, or lowest concentration exhibiting reduced RFI, was determined based on statistically significant reductions relative to the growth of the untreated control. For biofilm assays, 1 × 10^7^ bacteria/well were dispensed in RPMI medium with 10% HI-FBS and were incubated at 37°C under 5% CO_2_ for 24 h, after which the antibiotic was added to each well at the appropriate concentration, and results were compared with those for untreated controls.

For hydrogen peroxide (H_2_O_2_) assays, H_2_O_2_ (30%) (Fisher Scientific) was freshly diluted in RPMI medium –10% HI-FBS for each assay and was added to 1 × 10^7^ *Ma^Sm^* or *Ma^Rg^* planktonic bacteria/well or to 24-h biofilms for a final range of concentrations from 0.1 to 100 mM. RFI was quantified as described above. For pH assays, acidic pHs (5.5, 4.5, and 3.5) were obtained by diluting 7H9 broth–OADC with HCl. Acetic acid was diluted to 5, 2.5, or 1% in 7H9 broth–OADC.

### Macrophage culture and infection.

THP-1 cells (ATCC) were cultured as recommended by ATCC in RPMI 1640 medium with 2 mM l-glutamine–10% HI-FBS and 100 U/ml penicillin–100 μg/ml streptomycin at 37°C under 5% CO_2_. For differentiation to macrophage-like cells, THP-1 cells were added to 24-well tissue culture plates (Costar) in antibiotic-free medium and were treated with 10 ng/ml of phorbol 12-myristate 13-acetate (PMA) (Sigma) for 48 h to promote attachment. Monolayers were checked before each experiment, and cells were infected using a single-cell suspension of *Ma^Sm^* or *Ma^Rg^* with a multiplicity of infection (MOI) of 2.5 (2.5 bacteria to 1 macrophage) in RPMI medium with 20 mM HEPES (RH)–1 mg/ml human serum albumin (RHH) for 2 h at 37°C under 5% CO_2_. Each variant was opsonized with human serum for 30 min before incubation with THP-1 macrophages on a rotating platform for 30 min to ensure even infection, followed by stationary incubation for 90 min. After infection, macrophages were washed three times to remove extracellular or nonassociated bacilli before the addition of fresh RPMI medium with 10% HI-FBS and were either left untreated or treated with 100 μl/ml amikacin or 32 μl/ml azithromycin for 24 or 48 h.

For CFU counts, plates were removed after 2, 24, and 48 h, washed three times, and lysed with 0.25% SDS in phosphate-buffered saline (PBS) for 10 min in the presence of 50 μg/ml of DNase (Sigma) as described elsewhere (64). The lysate was then placed in 7H9 broth–OADC–0.5% Tween 80 with glass beads, pulse vortexed, serially diluted, and plated onto 7H10 agar. Agar plates were incubated at 37°C under 5% CO_2_ for a minimum of 3 days before enumeration of CFU.

For microscopy, PMA-treated THP-1 cells (4 × 10^5^) were plated onto 35-mm glass-bottom (no. 1.5) MatTek dishes (MatTek Corp., Ashland, MA) for 48 h prior to infection with opsonized mCherry-transformed *Ma^Sm^* or *Ma^Rg^* at an MOI of 2.5:1. At each time point, the cells were imaged with an Olympus FluoView FV10i confocal laser scanning microscope system (Olympus, Center Valley, PA). The mean number of infected macrophages on each MatTek plate was determined by counting ≥300 consecutive THP-1 cells per duplicate plate using phase-contrast and red fluorescence channels (64).

### Scanning electron and confocal microscopy.

For scanning electron microscopy, 72-h biofilms were rinsed using PBS and were fixed in glutaraldehyde, postfixed with osmium tetroxide, and dehydrated with ethanol as described previously (65). The samples were coated with gold-palladium using a sputter coater and were imaged using a Nova NanoSEM 400 system (FEI Co., Hillsboro, OR).

Confocal microscopy was performed using a Nikon A1R confocal system equipped with 60× (numerical aperture [N.A.] 1.4) and 100× (N.A. 1.45) oil immersion objective lenses on mCherry-transformed *Ma^Sm^* or *Ma^Rg^* 48-h biofilms, either alone or stained with FM 1-43 (Molecular Probes, Eugene, OR). Images were processed for presentation with Nikon Elements software (version 4.30.02).

### Statistical analysis.

Statistical analysis was performed in GraphPad Prism (version 5.0 for Windows; GraphPad Software, San Diego, CA, USA) using unpaired *t* tests or 2-way analysis of variance (ANOVA) for comparisons between data sets.

## Supplementary Material

Supplemental material
