# Supplementary material for: Mycobacterium abscessus Smooth and Rough Morphotypes Form Antimicrobial-Tolerant Biofilm Phenotypes but Are Killed by Acetic Acid
Source: Antimicrob Agents Chemother. 2018 Feb 23;62(3):e01782-17. doi: 10.1128/AAC.01782-17 (PMC5826145; doi:10.1128/AAC.01782-17)
Supplement: Supplemental material [file supp_62_3_e01782-17__index.html]

Supplemental material 

# Mycobacterium abscessus Smooth and Rough Morphotypes Form Antimicrobial-Tolerant Biofilm Phenotypes but Are Killed by Acetic Acid

## Supplemental material

- Supplemental file 1 -

  Fig. S1

  PDF, 141K
