# Supplementary material for: Mycobacterium abscessus Smooth and Rough Morphotypes Form Antimicrobial-Tolerant Biofilm Phenotypes but Are Killed by Acetic Acid
Source: Antimicrob Agents Chemother. 2018 Feb 23;62(3):e01782-17. doi: 10.1128/AAC.01782-17 (PMC5826145; doi:10.1128/AAC.01782-17)
Supplement: Supplemental material [file AAC.01782-17_zac003186955s1.pdf]

Supplementary Methods

THP-1 cells were plated and infected as described for confocal microscopy. Once infected, cells were treated with Amikacin (100µg/ml) or azithromycin (32µg/ml). Untreated cells for each morphotype served as controls. The infection was stopped at desired time points by washing the monolayers with warm RPMI 3 times to remove the extracellular bacteria. The monolayers were then with fixed with 4% paraformaldehyde in RPMI (Electron Microscopy Sciences, Hartfield, PA) for 15 minutes at room temperature and images taken at 24 and 48h following treatment. Nuclei were visualized using Syto9 (Molecular Probes, Eugene, OR) and images were captured using a Nikon A1R confocal system equipped with a 60x oil-immersion objective lens (N.A. 1.4). Image processing was performed Nikon Elements software (ver. 4.30.02). Maximum intensity projections of the image stacks were created and combined with a single DIC image from that stack (Supplemental Fig. 1). Nuclear signal was pseudocolored blue for enhanced contrast with the bacterial signal (red).

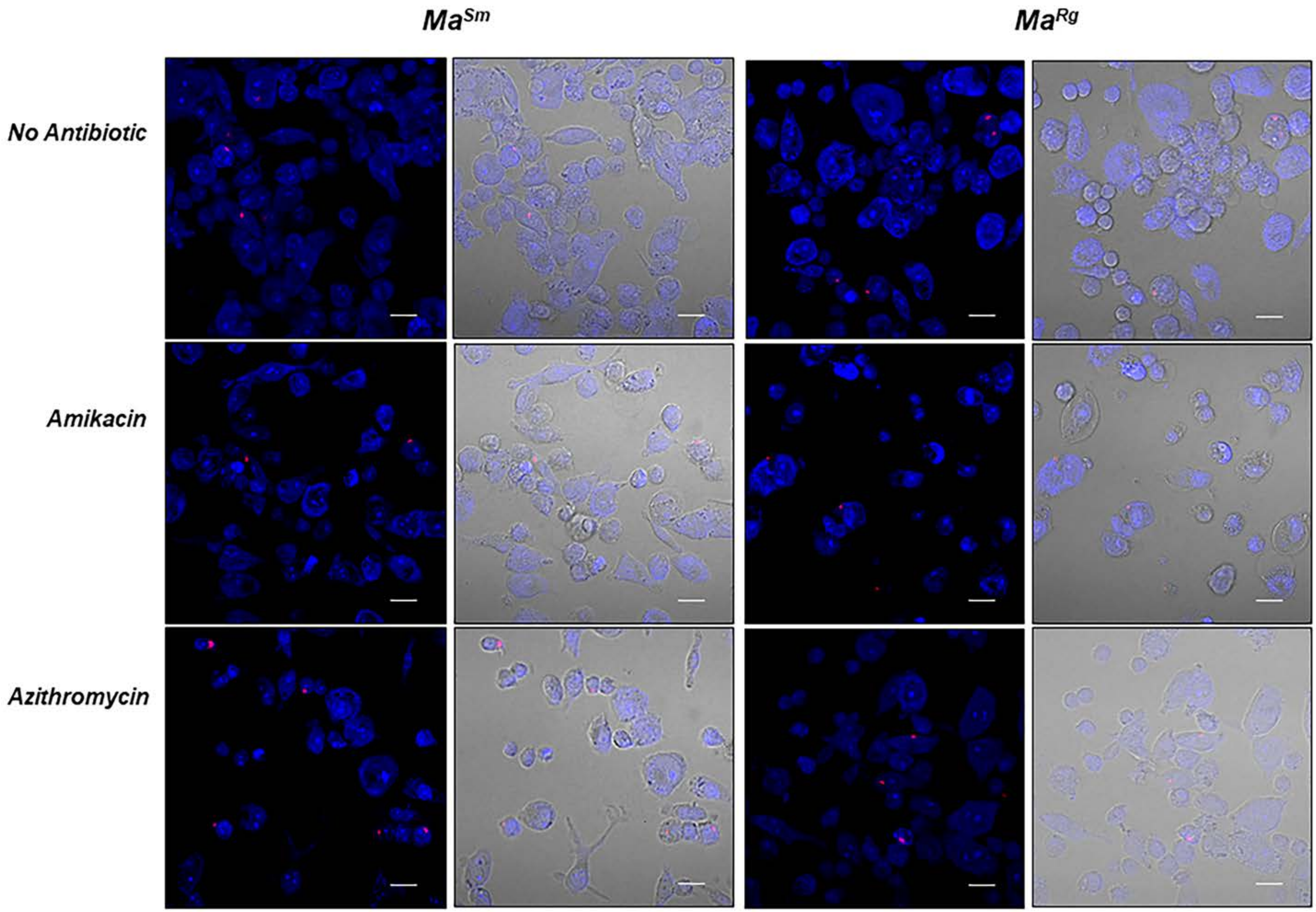
Ma^{Sm}
Ma^{Rg}
No Antibiotic
Amikacin
Azithromycin
